# Initial Studies of the Response of Rubber Tree Seedlings Treated with Saprobic Fungi from the Semiarid Region of Northeast Brazil to Anthracnose

**DOI:** 10.3390/plants11192477

**Published:** 2022-09-22

**Authors:** Marcela Pagoti Bergamini Lopes, Marcela Eloi Gomes, Gustavo da Silva Celin, Heloísa Noemi Bello, Rodney Lucio Pinheiro Henrique, Izabela Ponso Magalhães, Louyne Varini Santos, Leandro Tropaldi, Sergio F. Pascholate, Edson Luiz Furtado, Ana Carolina Firmino

**Affiliations:** 1 School of Engineering, São Paulo State University (Unesp), Ilha Solteira 15385-000, Brazil; 2 College of Agricultural and Technological Sciences, São Paulo State University (Unesp), Dracena 17900-000, Brazil; 3 Luiz de Queiroz College of Agriculture, University of São Paulo (USP), Piracicaba 13418-900, Brazil; 4 School of Agriculture, São Paulo State University (Unesp), Botucatu 18610-034, Brazil

**Keywords:** *Colletotrichum*, *Hevea brasiliensis*, control, resistance induction

## Abstract

Anthracnose, caused by fungi of the genus *Colletotrichum,* is present in the main areas where rubber trees (*Hevea brasiliensis*) are planted. Thus, considering that biological agents can be an alternative for disease control, the present study aimed to carry out initial studies to investigate the response of rubber tree seedlings inoculated with Colletotrichum and treated with saprobes fungi from the semiarid region of Northeast Brazil (*Curvularia eragrostidis*, *Memnoniella levispora*, *Myrothecium roridum* and *Phialomyces macrosporus*). Seedlings of the rubber tree clone RRIM600 were sprayed with biocontrol agents as preventive and curative treatments seven days before and after *C. tamarilloi* inoculation, respectively. Assessments included plant response to disease expression based on the percentage of symptomatic area on treated leaves, percentage of graft death, and percentage of apical death in seedlings 30 days after inoculation with *C. tamarilloi*. In addition, the enzymes peroxidase and phenylalanine ammonia lyase (PAL) had their activity quantified by their association with plant resistance to pathogens. The fungus *C. eragrostidis* had the best result in controlling anthracnose when applied as a preventive treatment, showing 10% less disease than the untreated plant. The same was observed for the fungus *P. macrosporus* when used in the curative form. These fungi also reduced the graft death. In these cases, PAL activity was higher and may be linked to the induction of resistance against the pathogen. The peroxidase activity was not expressive for treatments with saprobic fungi in the periods studied. Therefore, among the tested fungi, *C. eragrostidis* and *P. macrosporus* are promising for the control of anthracnose, deserving further studies.

## 1. Introduction

The rubber tree (*Hevea brasiliensis*) is a species from the Amazon region, which belongs to the family Euphorbiaceae. This species has great economic importance due to its production of latex, a source of natural rubber [1]. Phytosanitary problems, such as those caused by anthracnose, can limit the increase in crop productivity. This disease that has been affecting this crop, in its severe form, causes defoliation of the plants, descending drought of the branches and death of the apical buds, negatively interfering in the production of latex by the plant [2].

There is evidence of rubber tree clones resistant to anthracnose; however, they were not planted in the field yet [3]. Thus, the control of the disease is carried out mainly in the application of fungicides in nurseries due to the ease of application, due to the size of the plant [4,5]. In the field, some limitations of this method are the uniformity of the tree canopy and the stage of the disease, which is often very advanced, being economically unfeasible [6]. The data bank on agrochemical and related products registered at the Brazilian Ministry of Agriculture [7] shows that products of the strobilurin and triazole groups are the basis for *C. gloeosporioides* control in rubber trees. These products act systemically on the plants and, if applied frequently, can lead to the selection of fungal populations resistant to their active principles [8].

The occurrence of more than one species of anthracnose-causing fungus parasitizing the same host in a given area should also be taken into account, as it may interfere with the chemical control of the disease. This is because *C. acutatum*, *C. boninense*, *C. capsici*, *C. coccodes*, and *C. gloeosporioides* have been reported to have differentiated sensitivity to fungicides, azoxystrobin abse, carbendazim, thiabendazole, and tebuconazole [9].

As an alternative to the use of chemical pesticides and to the lack of resistant materials in the market, studies on resistance induction have emerged recently. According to Kuhn [10], this strategy consists in adopting abiotic or biotic agents of low environmental impact to stimulate the plant defense system and mechanisms, triggering a response of resistance to diseases. Among such agents are fungi and bacteria, which not only induce resistance in the plant, but can also act as antagonists to pathogenic microorganisms [11].

Following this line of thought, bioprospecting studies of saprobes fungi from the Brazilian northeast semiarid region have been explored, since these fungi have the ability to remove nutrients from dead tissue, resistance to water restriction and competitiveness with other microorganisms, in addition to secreting pectinases and oligogalacturonides, thus being able to activate the plant’s defense responses [12,13,14]. For example, in the control of *Sclerotinia sclerotiorum* in soybean, *Myrothecium* sp. showed potential for use as a biocontroller of the disease. In the case of sorghum, among the 16 fungi used (*Curvularia inaequalis*, *Gonytrichum macroladum*, *Memnoniella levispora*, *Pithomyces chartarum*, *Periconia hispidula*, *Phaeoisaria clematidia*, *Dictyochaeta heteroderae*, *Sarcopodium circinatum*, *Periconia byssoides*, *Moorella speciosa*, *Stachybotrys chartarum.*, *Pseudobotrytis terrestrials*, *Stachybotrys globosa* and *Gonytrichum clamydosporium*), only *C. inaequalis* was able to control *Colletotrichum sublineolum*, significantly increasing the activities of peroxidases, chitinases and β-1,3-glucanases in plants [12,13,14].

In the case of forest essences, studies are scarcer. A study points to the prospective action of saprobes fungi from the semiarid region of Northeastern Brazil in the activation of latent resistance mechanisms against *A. psidii* in seedlings of eucalyptus clones. In this case *Phialomyces macrosporus* is an ideal biotic inducer for rust, since it inhibited the germination of urediniospores and increased the activity of PAL, peroxidase and β-1,3-glucanase. Other fungi that also show promising results are *C. eragrostidis*, which reduced the severity of rust and induced the rooting of cuttings; *Stachybotrys chartarum*, which reduced rust severity and increased β-1,3-glucanase activity, and *C. inaequalis*, which led to increased PAL activity [15].

Thus, the objective of this work was to start studying the response of rubber tree seedlings to anthracnose treated with fungi belonging to Brazilian biodiversity, in this case, saprobes from the semi-arid region of the Northeast. In addition, the activity of peroxidase and PAL in plants treated with these fungi was quantified, since these enzymes are known to have post-formed biochemical resistance mechanisms in the plant. With this, it will be possible to deepen the studies with these fungi in the rubber tree culture in order to find an alternative and sustainable method for the management of this disease.

## 2. Materials and Methods

### 2.1. Fungal Isolate and Rubber Tree Clones Used in the Study

To conduct the study, a *Colletotrichum tamarilloi* isolate (CH09) from rubber tree, which was stored at the forest pathology fungal collection of FCAT/UNESP, was molecularly characterized by sequencing of part of the rDNA regions ITS-5.8S rDNA (MW031267), part of β-Tubulin region (OK258095), part of glyceralde-hyde-3-phosphate dehydrogenase (GAPDH) gene (OK258094) and part of calmodulin (CAL) gene (OK258093) [16,17,18]. The used inoculum was stored in oil and was activated in oat medium, at 25 ± 1 °C and continuous photoperiod, for seven days. The colony of the isolate was washed in distilled water and the obtained suspension was filtered through sterile gauze, quantified in a Neubauer chamber and adjusted to 10^5^ conidia/mL.

The saprobic fungi used in this study were collected and isolated from the semi-arid region of Northeast Brazil and are deposited at CMB (Culture Collection of Microorganisms of Bahia), which is located at the State University of Feira de Santana, Bahia State, and is certified by CGEN (Genetic Heritage Management Council). *Curvularia eragrostidis*, *M. levispora*, *M. roridum* and *P. macrosporus* were chosen for their good results in studies on induction of resistance to rust in eucalyptus [15]. These fungi were cultured in oat medium at 25 ± 1 °C and with an alternating photoperiod, as described by Pierozzi [15]. The colonies of each isolate were washed in sterile distilled water and the obtained suspension was filtered in sterile gauze, quantified in a Neubauer chamber, and adjusted to 10^5^ conidia/mL.

The clone RRIM 600 was employed in the present study because it is most planted in São Paulo State and is susceptible to the disease. Seedlings (aged around six months old) were obtained from a commercial nursery.

### 2.2. Response of Rubber Tree Seedlings to Colletotrichum Using Saprobic Fungi from the Semi-Arid Region of Northeast Brazil

To verify the response to anthracnose from rubber tree seedlings treated with the saprobic fungi, both the curative effect and the protective effect of these fungi on the plant were assessed. The fungi were sprayed until runoff (approximately 100 mL/plant) on seedlings of the rubber tree clone RRIM600.

To investigate the plant response to the curative treatment, the pathogen was inoculated seven days before the spraying of saprobic fungi. For preventive effect assessment, the pathogen was inoculated seven days after the application of saprobic fungi. The experiment consisted in five treatments to study the preventive effect and five treatments to study the curative effect, which were based on: (1) plants sprayed with *C. tamarilloi* alone; (2) plants sprayed with *M. roridum* (Isolate 03/10); (3) plants sprayed with the control agent *P. macrosporus* (isolate 37/06); (4) plants sprayed with the control agent *C. eragostilis* (Isolate 47/06); and (5) plants sprayed with the control agent *M. levispora* (isolate 33/08). The experiment was carried out in a completely randomized design. Ten replicates per treatment were adopted. Each replicate consisted of one single plant. The experiment was conducted twice.

Thirty days after the spraying of control agents, the percentage area showing symptoms of *C. tamarilloi* was evaluated based on photographs of five leaves per seedling analyzed with the application Leaf Doctor [19]. The obtained results underwent analysis of variance and means were compared according to Tukey’s test at 5% probability level, using the software Sisvar [20]. The percentage of plant death and apical death were also evaluated at this period.

### 2.3. Quantification of the Activity of Enzymes Peroxidase and Phenylalanine Ammonia Lyase (PAL)

For these analyses, an apical leaf was collected from each treated seedling. To detect the enzyme activity, protein extract was obtained and quantified according to the methodology of Bradford [21]. Crude extract from each treatment was used to determine all enzymes. The activity of peroxidases was analyzed based on the methodology described by Boava et al. [22] and expressed as absorbance units/min/mg/protein. The activity of PAL was analyzed according to [15] and measured based on its expression as ng/mL trans-cinnamic acid/g protein/h.

All enzymatic assays were conducted in triplicate for each treatment. The obtained values underwent analysis of variance and means were compared according to Skott-Knott test at 5% probability level, using the software Sisvar [20].

## 3. Results

**Figure 1:**
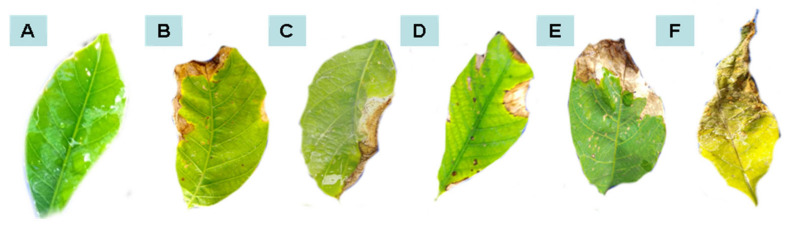
Leaf area affected by anthracnose after curative treatments with water (**A**), *Colletotrichum* alone (**B**), *P. macrosporus*—Isolate 37/06 (**C**), *C. eragrostidi*—Isolate 47/06 (**D**), *M. levispora*—Isolate 33/08 (**E**), and *M. roridum*—Isolate 03/10 (**F**).

**Figure 2:**
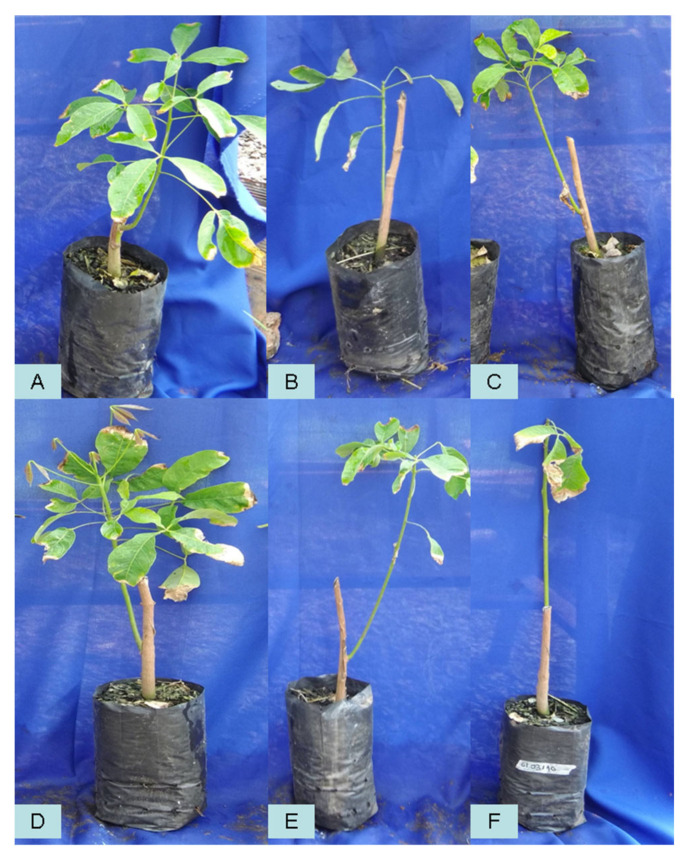
Seedlings of the rubber tree clone RRIM600 after curative treatments with water (**A**), *Colletotrichum* alone (**B**), *P. macrosporus*—Isolate 37/06 (**C**), *C. eragrostidi*—Isolate 47/06 (**D**), *M. levispora*—Isolate 33/08 (**E**), and *M. roridum*—Isolate 03/10 (**F**).

**Figure 3:**
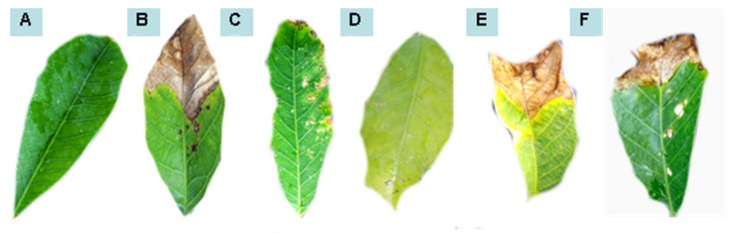
Leaf area affected by anthracnose after preventive treatments with water (**A**), *Colletotrichum* alone (**B**), *P. macrosporus*—Isolate 37/06 (**C**), *C. eragrostidi*—Isolate 47/06 (**D**), *M. levispora*—Isolate 33/08 (**E**), and *M. roridum*—Isolate 03/10 (**F**).

**Figure 4:**
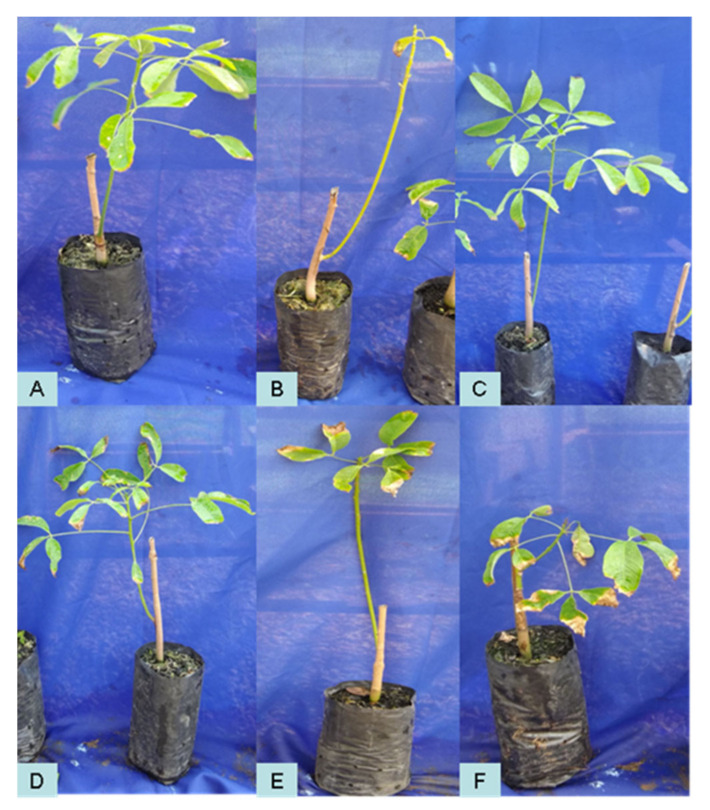
Seedlings of the rubber tree clone RRIM600 after preventive treatments with water (**A**), *Colletotrichum* alone (**B**), *P. macrosporus*—Isolate 37/06 (**C**), *C. eragrostidi*—Isolate 47/06 (**D**), *M. levispora*—Isolate 33/08 (**E**), and *M. roridum*—Isolate 03/10 (**F**).

The rubber tree seedlings behaved differently when saprobic fungi were applied either as preventive treatment or as curative treatment. The fungus *C. eragrostidis* led to less disease severity when applied as preventive treatment. Similarly, when exposed to *P. macrosporus*, the plant demonstrated less disease after the curative treatment (Table 1 and Table 2). Figure 1, Figure 2, Figure 3 and Figure 4 illustrate the appearance of leaves and canopy of seedlings treated with these fungi, compared to seedlings that did not receive any type of treatment.

The activity of PAL was more expressive in the treatments that led the plant to demonstrate a positive response, i.e., a lower diseased leaf area percentage (Table 3 and Table 4). In general, the activity of peroxidase in treated plants could not be associated with a positive response from the plant, i.e., less disease occurrence (Table 5 and Table 6).

## 4. Discussion

The differentiated behavior, resulting from the form of application of saprobes fungi in the plants, has already been reported. Pierozzi [15], who also worked with these same saprobic fungal isolates, reported a difference in the behavior of eucalyptus seedlings to diverse treatments, either preventive or curative treatments. Testing the effect of saprobic fungi on rust severity, that author noticed that plants susceptible to *A. psidii* had lower rust intensity when they received preventive inoculation with *C. eragrostidis* and curative treatment with *C. inaequalis*. According to that author, for eucalyptus, the preventive action of these saprobic fungi is more effective than their curative effect, which was not observed in the present study since the fungi that were effective in controlling the disease had similar diseased leaf area percentages.

It must be highlighted that Pierozzi [15] also observed that some fungi stimulated *A. psidii* germination. In the present study, the affected leaf area increased with *M. levispora*—Isolate 33/08 as preventive treatment and with *M. roridum*—Isolate 03/10 as curative treatment. Thus, two hypotheses can be considered: the first one is that these fungi can produce substances that favor *Colletotrichum* development, and the second hypothesis predicts that *M. levispora* and *M. roridum* are potential pathogens to the rubber tree, since structures typical of these fungi were found in treated leaves, which must be further investigated. On the other hand, Rocha et al. [23] reported that an isolate of the genus *Myrothecium* sp., from the endophytic microfauna of rubber tree leaves, was effective in controlling *Microcyclus ulei*, the causative agent of leaf blight in rubber trees. Such information indicates that isolates of the same fungal genus or species may interact differently with the plant to which they are applied, that is, they may or may not cause the plant to express its resistance mechanisms. The explanation for this behavior can be based on an old theory, the Gene-to- Gene Flower Theory, which, although not universal, provides a model that points to the one-to-one relationship between attack and defense genes, respectively, of the pathogen. and in the host. With this, it is possible to understand how a microorganism develops attack mechanisms that allow “breaking” the non-host resistance [24], and thus become phytopathogenic, as can be the case of the fungi *M. levispora* and *M. roridum* with the rubber tree. The Saprobic fungi have been tested for phytopathogen control, showing to be interesting alternatives in the management of diseases. In addition to the previously mentioned cases of the use of these fungi in the control of *Sclerotinia sclerotiorum* in soybean and sorghum [12,13,14], there are more indications of the bioprospecting of these fungi in other cultures. For example, Solino et al. [25] demonstrated that filtrates of *C. inaequalis*, *Pseudobitritis terrestris*, *Memnomiella echinata* and *C. eragrostidis* are effective in increasing phytoalexins in bean, sorghum and soybean, which can help protect the plant against the attack of diverse pathogens, since this compound is part of the plant defense [26]. In grape leaves, filtrates of the saprobic fungi *C. inaequalis* and *Stachybotrys globosa*, especially at higher concentrations, were efficient in reducing mildew and *Isariopsis* spot, as well as mildew severity in grape clusters. However, those authors highlighted that such efficiency was lower under conditions of more favorable climate to the pathogen development and high inoculum pressure. Therefore, filtrates are not recommended for mildew control in grapevines, either for leaves or for clusters, in areas of high inoculum pressure and in periods of median temperatures and high humidity [27]. Such notice can be extended to the present study, which was conducted under optimal conditions to the pathogen development, especially concerning the age of the used plant, initial development stage, and its greater susceptibility to the disease. Thus, these fungi should be tested in older plants.

Graft dead and apical bud death were caused by *Colletotrichum* since signs of acervuli and orange mucilage were found. This type of symptom may cause direct losses to the seedling producer because the plant will die or delay its development for new sprout production. According to Noal et al. [28], in general, approximately 4.99% grafts are lost; moreover, during the handling of grown grafted seedlings, like: decapitation for rootstock shoot removal, digging and preparation of beds, there is more than 3.54% seedling loss. Such losses of grafted seedlings added to losses those caused by diseases, like in the present study that reached up to 20%, may result in huge losses to the farmer. Thus, Noal et al. [28], as well as the current results, evidenced the major importance of maintaining a clone garden where buds are originated to be used in grafting, preventively spraying fungicides at every 40 days and pruning in the correct period to timely obtain healthy clones of excellent quality.

The PAL enzime presented greater activity in plants that had less anthracnose symptoms, which can be related to the production of the phenolic compounds present in the formation of esters, flavonoids and lignins used in the plant defense [26]. For rubber trees, there are reports of accumulation of some compounds, e.g., phenolic compounds, under attack from *P. palmivora* [29]. Magalhães et al. [30] studied different clones inoculated with *Colletotrichum* and observed the resistance of rubber trees to anthracnose to have a strong connection to the production of lignins and phenols, which are related to the activity of PAL. Similar results were obtained by Pierozzi [15] in eucalyptus plants treated with saprobic fungi from the semi-arid region of Northeast Brazil. According to this authors, plants inoculated with *P. macrosporus* and *C. inaequalis* were highlighted as the major activators of PAL activity, presenting high rates in the evaluations at 96 and 72 h after inoculation, respectively.

Differently from PAL, the peroxidase enzyme activity in the treated plants, it could not be associated with a positive plant response, probably due to the period of its evaluation. Studies carried out with *Citrus aurantium* indicated that there is a drop in the activity of some resistance-related enzymes a few hours after receiving the chitin elicitor. This finding was correlated with the ability of plants to quickly restore their normal metabolism, which is a possible explanation for the low activity of most enzymes evaluated within 96 h [31]. Even so, there are studies that point to the high activity of this enzyme in rubber trees and eucalyptus when plants are inoculated with *Colletotrichum* and *Ralstonia solanacearum*, respectively, in the same time periods evaluated in the present work, but in both cases other biological agents were used of control, *Trichoderma harzianum* and *Bacillus subtilis*, which were applied in the soil [32,33]. These facts may also explain the different behavior of the peroxidase enzyme, which may be linked to the way the treatment is applied and the microorganisms used, as this fact influences the plant’s response in its induction. The importance of time, frequency, and application method of application of the control agent can influence the improvement of results according to [34]. *M. roridum* was the only applied saprobic isolate that had an effect on the activity of peroxidase, but only in the first evaluation. As evidenced by the affected leaf areas shown in Table 2 and in the figures, this fungus stimulated a negative response from the plant, which had greater leaf area affected by *Colletotrichum*, especially after the curative treatment. This can explain the increased activity of peroxidase when *M. roridum* was applied, since the plant had to activate its defense mechanisms.

## 5. Conclusions

The fungus *P. macrosporus* controlled the disease when used as a curative treatment, while *C. eragrostidis* had the best result in controlling anthracnose when applied as a preventive treatment. Regarding the enzymes studied, PAL activity was more expressive after treatments that reduced the percentage of diseased leaf area, while the activity of peroxidase in the periods evaluated could not be associated with the occurrence of the disease in plants that received preventive or curative treatments in this case. Thus, among the tested fungi, *C. eragrostidis* and *P. macrosporus* showed promise for the control of anthracnose in rubber trees, deserving studies on larger scales, including clonal garden and field. If these fungi show promise at all stages, they could be produced on a larger scale, constituting an alternative for controlling anthracnose in rubber trees. Therefore, this work opens opportunities for further studies with these fungi.

## Figures and Tables

**Table 1:** Percentage of diseased leaf area, graft dead and apical bud death in seedlings after curative treatment *.

Treatments	Diseased Leaf Area (%)	Graft Dead (%)	Apical Bud Death (%)
Just *C. tamarilloi*	19.37 bc	20	0
*C. tamarilloi + M. roridum*	31.46 c	0	20
*C. tamarilloi + M. levispora*	14.06 b	20	20
*C. tamarilloi + P. macrosporus*	9.31 a	0	0
*C. tamarilloi + C. eragrostidis*	14.46 b	20	0
CV	45.21	-	-

* Means of two experiments. CV, coefficient of variation transformed into square root—SQRT (Y). Means followed by the same lowercase letters in the columns are not significantly different (Tukey test; *p* < 0.05).

**Table 2:** Percentage of diseased leaf area, graft dead and apical bud death in seedlings after preventive treatment *.

Treatments	Diseased Leaf Area (%)	Graft Dead (%)	Apical Bud Death (%)
Just *C. tamarilloi*	17.37 bc	20	10
*M. roridum + C. tamarilloi*	16.65 b	20	20
*M. levispora + C. tamarilloi*	20.71 c	20	0
*P. macrosporus + C. tamarilloi*	15.57 b	20	10
*C. eragrostidis + C. tamarilloi*	7.11 a	0	10
CV	50.55	-	-

* Means of two experiments. CV, coefficient of variation transformed into square root—SQRT (Y). Means followed by the same lowercase letters in the columns are not significantly different (Tukey test; *p* < 0.05).

**Table 3:** Activity of the enzyme PAL in rubber tree seedlings after curative treatments with saprobic fungi.

Treatments	Activity of PAL (ng/mL Trans-Cinnamic Acid. g protein^−1^·h^−1^)		
	7 Days	14 Days	21 Days
Just *C. tamarilloi*	0.710 A ab	0.850 Bb	0.640 Aa
*C. tamarilloi + M. roridum*	0.630 A a	0.810 Bb	0.760 Bb
*C. tamarilloi + M. levispora*	0.820 B b	0.650 Aa	0.680 Aa
*C. tamarilloi + P. macrosporus*	0.810 B ^ns^	0.900 C	0.890 B
*C. tamarilloi + C. eragrostidis*	0.870 B ^ns^	0.840 B	0.830 B
CV	42.1	47.2	31.7

CV, coefficient of variation of the data transformed into square root—SQRT, (Y). Means followed by the same uppercase letters in the columns and the same lowercase letters on the rows are not significantly different. ^ns^ non-significant test on line (Tukey test; *p* < 0.05).

**Table 4:** Activity of the enzyme PAL in rubber tree seedlings after preventive treatments with saprobic fungi.

Treatments	Activity of PAL (ng/mL Trans-Cinnamic Acid. g protein^−1^·h^−1^)		
	7 Days	14 Days	21 Days
Just *C. tamarilloi*	0.720 A ^ns^	0.610 A	0.770 A
*M. roridum* + *C. tamarilloi*	0.880 B ^ns^	0.910 C	1.000 B
*M. levispora* + *C. tamarilloi*	0.740 Aa	0.910 Cb	0.980 Bb
*P. macrosporus* + *C. tamarilloi*	1.060 Cb	0.780 Ba	0.860 ABab
*C. eragrostidis + C. tamarilloi*	0.930 C ^ns^	1.080 D	1.033 B
CVT	23.1	18.4	27.4

CVT, coefficient of variation of the data transformed into square root—SQRT, (Y). Means followed by the same uppercase letters in the columns and the same lowercase letters on the rows are not significantly different. ^ns^ non-significant test on line (Tukey test; *p* < 0.05).

**Table 5:** Activity of the enzyme peroxidase in rubber tree seedlings after curative treatments with saprobic fungi.

Treatments	Activity of Peroxidase (mmol·min^−1^·mg^−1^ protein)		
	7 Days	14 Days	21 Days
Just *C. tamarilloi*	0.8030 Bb	0.4270 a	0.4500 a
*C. tamarilloi + M. roridum*	0.3975 A ^ns^	0.4985	0.3780
*C. tamarilloi + M. levispora*	0.4984 A ^ns^	0.4765	0.3800
*C. tamarilloi + P. macrosporus*	0.5256 Ab	0.3833 a	0.3210 a
*C. tamarilloi + C. eragrostidis*	0.3544 Aa	0.6513 b	0.5200 b
CV	37.5	38.8	31.2

CV: coefficient of variation of the data transformed into square root—SQRT, (Y). Means followed by the same uppercase letters in the columns and the same lowercase letters on the rows are not significantly different. ^ns^ non-significant test on line (Tukey test; *p* < 0.05).

**Table 6:** Activity of the enzyme peroxidase in rubber tree seedlings after preventive treatments with saprobic fungi.

Treatments	Activity of Peroxidase (mmol·min^−1^·mg^−1^ protein)		
	7 Days	14 Days	21 Days
Just *C. tamarilloi*	0.854 Cb	0.555 BCa	0.520 BCa
*M. roridum* + *C. tamarilloi*	0.764 C ^ns^	0.840 C	0.800 C
*M. levispora* + *C. tamarilloi*	0.162 A ^ns^	0.286 A	0.240 A
*P. macrosporus* + *C. tamarilloi*)	0.501 B ^ns^	0.560 B	0.520 BC
*C. eragrostidis + C. tamarilloi*	0.241 A ^ns^	0.249 A	0.120 A
CV	38.1	41.0	35.2

CV: coefficient of variation of the data transformed into square root—SQRT, (Y). Means followed by the same uppercase letters in the columns and the same lowercase letters on the rows are not significantly different. ^ns^ non-significant test on line (Tukey test; *p* < 0.05).

## Data Availability

The data presented in this study are available on request from the corresponding author. The data are not publicly available due to be part of a bigger project.
